# Gorge Motions of Acetylcholinesterase Revealed by Microsecond Molecular Dynamics Simulations

**DOI:** 10.1038/s41598-017-03088-y

**Published:** 2017-06-12

**Authors:** Shanmei Cheng, Wanling Song, Xiaojing Yuan, Yechun Xu

**Affiliations:** CAS Key Laboratory of Receptor Research, Drug Discovery and Design Center, Shanghai Institute of Materia Medica, Chinese Academy of Sciences (CAS), Shanghai, 201203 China

## Abstract

Acetylcholinesterase, with a deep, narrow active-site gorge, attracts enormous interest due to its particularly high catalytic efficiency and its inhibitors used for treatment of Alzheimer’s disease. To facilitate the massive pass-through of the substrate and inhibitors, “breathing” motions to modulate the size of the gorge are an important prerequisite. However, the molecular mechanism that governs such motions is not well explored. Here, to systematically investigate intrinsic motions of the enzyme, we performed microsecond molecular dynamics simulations on the monomer and dimer of *Torpedo californica* acetylcholinesterase (*Tc*AChE) as well as the complex of *Tc*AChE bound with the drug E2020. It has been revealed that protein-ligand interactions and dimerization both keep the gorge in bulk, and opening events of the gorge increase dramatically compared to the monomer. Dynamics of three subdomains, S3, S4 and the Ω-loop, are tightly associated with variations of the gorge size while the dynamics can be changed by ligand binding or protein dimerization. Moreover, high correlations among these subdomains provide a basis for remote residues allosterically modulating the gorge motions. These observations are propitious to expand our understanding of protein structure and function as well as providing clues for performing structure-based drug design.

## Introduction

Acetylcholinesterase (AChE), a serine protease, widely distributed at neuromuscular junctions and cholinergic brain synapses, plays a pivotal physiological role by rapid hydrolysis of the neurotransmitter, acetylcholine (ACh), into acetate and choline^1, 2^. Accordingly, AChE has been extensively studied. In particular, it has been the prime target of the first generation of anti-Alzheimer’s drugs, as well as insecticides and nerve agents, and there is continued interest in discovering novel AChE inhibitors^3–9^.

**Figure 1:**
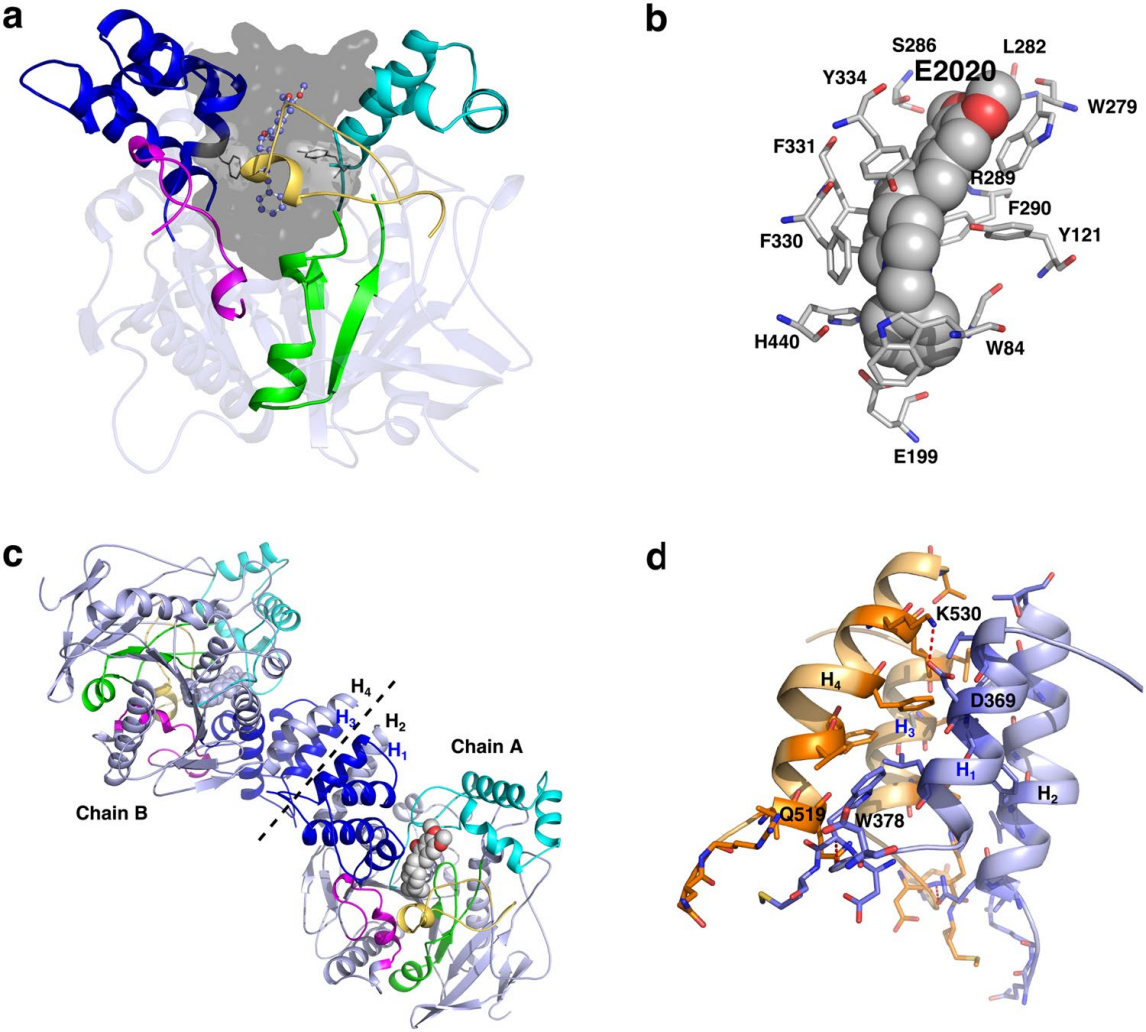
Cartoon representation of *Tc*AChE in complex with E2020 in a monomer and dimer. The crystal structure of *Tc*AChE in complex with E2020 (pdb code: 1eve) was used. (**a**) The active-site gorge of *Tc*AChE is shown as a grey molecular surface. The bound E2020 is shown as ball-and-sticks. Two “bottleneck” residues, Y121 and F330, are shown as sticks. Five subdomains surrounding the active-site gorge are highlighted with yellow (the Ω-loop, residues 67–94), green (S1, residues 114–150), cyan (S2, residues 225–296), blue (S3, residues 324–400), and magenta (S4, residues 428–450). (**b**) Interactions between 12 residues (sticks) and E2020 (spheres), calculated by the program LigPlot^50^. (**c**) A dimer of *Tc*AChE-E2020. The subdomains (Ω-loop, S1, S2, S3 and S4) are shown with same colors as they are in panel a. (**d**) The dimer interface is mainly constructed by four helices: H_1_ and H_2_ from chain A, and H_3_ and H_4_ from chain B. H_1_ or H_3_ (residues 361–381) is a helix from the subdomain S3, while H_2_ or H_4_ (residues 514–535) is a C-terminal helix. Inter-chain interacting residues, predicted by LigPlot, are shown as sticks. Hydrogen bonds are shown as red lines.

The catalytic rate of AChE on ACh is extremely fast (~10^9^ M^−1^·s^−1^), approaching the diffusion limit in substrate association and dissociation^10, 11^. However, crystal structures revealed that its active site including the catalytic triad is not easy to access^12^ as it is buried at the bottom of a deep (~20 Å) and narrow (~5 Å) gorge lined with multiple conserved aromatic residues (Fig. 1)^3, 4^. Much effort has been made to rationalize this paradox of, on the one hand, high catalytic activity, and on the other, a well-defined, rigid structure with a deep and narrow active-site gorge. One popular hypothesis is that multiple access pathways to the active site are propitious to the quick binding/release of the substrate as well as the product. To test this idea, a series of theoretical calculations relating to AChE have been carried out, including classic molecular dynamics (MD) simulations, Brownian dynamics simulations, multiple copy samplings, steered molecular dynamics simulations (SMD), and hybrid quantum mechanics/molecular mechanics (QM/MM) ﻿simulations﻿^13–26^. Besides the main exit along the long and narrow gorge of the enzyme, alternative pathways such as the “back-door” and the “side-door” have been successfully captured in simulations^14–16^. The “back-door” is generated as a result of concerted movement of the side-chains of W84, V129 and G441 (referring to residues of *Torpedo californica* AChE (*Tc*AChE)), and its transient opening provides a channel connecting the catalytic site with the bulk solvent^14^. The “side-door” is located at a distance of ~15 Å from the catalytic residue S200 and is approximately perpendicular to the gorge entrance composed of residues from the Ω-loop^15, 16^. Multiple copy samplings of the traffic of seven ligands within the enzyme found that different pathways may exist for different ligands to travel^17^. For small ligands such as ammonium or methane, exit from the gorge occurred via several routes; whereas the larger polar ligands methylammonium and acetic acid left the binding site solely via the main gorge. It was also found that the bulkiest ones, tetramethylammonium and neopentane, as well as the smaller acetate ion remained trapped inside the active site. Multiple dynamics simulations on the positively charged thiocholine, a mimic of the native product, showed that a majority of the ligands exited from the catalytic site via the “back-door”, some were retained at the active site, and very few were released through the “side-door” or the main gorge^18^. Recently, simulations of apo and Soman-adducted forms of human AChE (hAChE) showed that the substrate and product could utilize two different pathways for entry and exit in the apo form of AChE^19^. The main gorge has proved to be responsible for substrate selectivity, according to comparisons of MD simulations of hAChE and human butyrylcholinesterase (BChE)^13^. In conclusion, results from most simulations agree that the substrates as well as inhibitors enter into the catalytic site via the main gorge, while the products can exit via multiple pathways.

To arrive at the active site via the main gorge, a key step for the substrate is to cross over the “bottleneck” which is mostly contributed by F330 and Y121 in the middle of the main gorge (Fig. 1). “Breathing” motions that enlarge the gorge radius transiently, especially at the location of the “bottleneck”, are essential due to the observation that the narrowest size of this portion only allows one water molecule to pass through^18, 20–22, 27–29^. Our previous SMD simulations on the traffic of inhibitors such as Huperzine A and E2020 along the main gorge indicated that the opening of the “bottleneck” was a prerequisite^20, 21^. A 10-ns MD simulation on mouse AChE by Tai *et al*. found the minimal distance between F330 and Y121 correlated with the gorge radius^22^. Our previous MD simulations combined with multiple X-ray crystal structures of *Tc*AChE also revealed that the flexibility of F330 was the highest among 14 aromatic residues lining the gorge^30^, supposedly increasing the chance to break the “bottleneck”. However, the open state of the main gorge is rare. In an early study, using a 2.4 Å probe to model acetylcholine, only ~2.4% of the total 750 ps was detected as opening time^28^. Moreover, large conformational changes of the backbone of the entire enzyme, in particular for the region of the main gorge or other ligand-traffic pathways, could hardly be seen in previous, relatively short MD simulations. Therefore, the molecular mechanism that governs the “breathing” motions and factors regulating such motions are not yet clear, although it is well known that such “breathing” motions are necessary for the entry of bulk ligands into the catalytic site.

Additionally, almost all simulations were performed on the monomer of the enzyme; however, the biologically functional form is a dimer for *Tc*AChE and a tetramer for human AChE. It is well accepted that protein dynamics is a complex process, and not only the gorge residues but also other factors could have an influence on motions of the gorge through allosteric modulation. It has been shown that a ligand binding or a residue mutation at an allosteric site, coupled to an allosteric network based on water-mediated contacts, can lead to a drastic change in the map of signal transmission and protein activity^31–34^. To investigate the microscopic underpinnings of intramolecular signaling on the gorge motion, in this study, conventional MD simulations were performed on the monomer as well as the dimer of *Tc*AChE in an apo form and its complex with the inhibitor E2020, which has been approved by the FDA for treatment of Alzheimer’s disease (Fig. 1). First of all, we conducted a 1-μs MD simulation on the monomer *Tc*AChE, without adding any biased forces so as to explore the intrinsic features of “breathing” motions of *Tc*AChE and to evaluate the contribution of each subdomain surrounding the active-site gorge to motions of the gorge on the microsecond timescale. To the best of our knowledge, it is the longest sampling for this protein at the present time. A comparable simulation of a *Tc*AChE monomer in complex with E2020 was performed in parallel, so that the inner influence of the bound inhibitor on the gorge motion could be investigated. Moreover, to understand the transfer of allosteric modulation from the outside to the inside, MD simulations of the dimer of *Tc*AChE in an apo form and its complex with E2020 were performed, considering that the dimer of *Tc*AChE (G_2_) is the biologically functional form in electric organ tissue^35^. An important outcome of our study is that the “breathing” motions of the active-site gorge in four different states are not only observed by the examination of the minimal gorge radius, but also elucidated by the application of dynamic correlation analysis to dissect the enzyme into subdomains, residues and dynamic transmission pathways, allowing connections to be made between these dynamics and the variations of the minimal gorge radius. As a result, the dynamics of three mobile subdomains S3 (residues 324–400), the Ω-loop (residues 67–94) and S4 (residues 428–450) are shown to exert predominant effects on gorge motions (Fig. 1a), while the binding of inhibitor as well as protein dimerization can change the dynamic patterns of these subdomains and thereby affect the gorge motions. In addition, allosteric modulation of the gorge motions by the non-gorge residues as well as the dimerization interaction regions is also discussed.

## Results and Discussion

### The minimal radius of the active-site gorge

**Figure 2:**
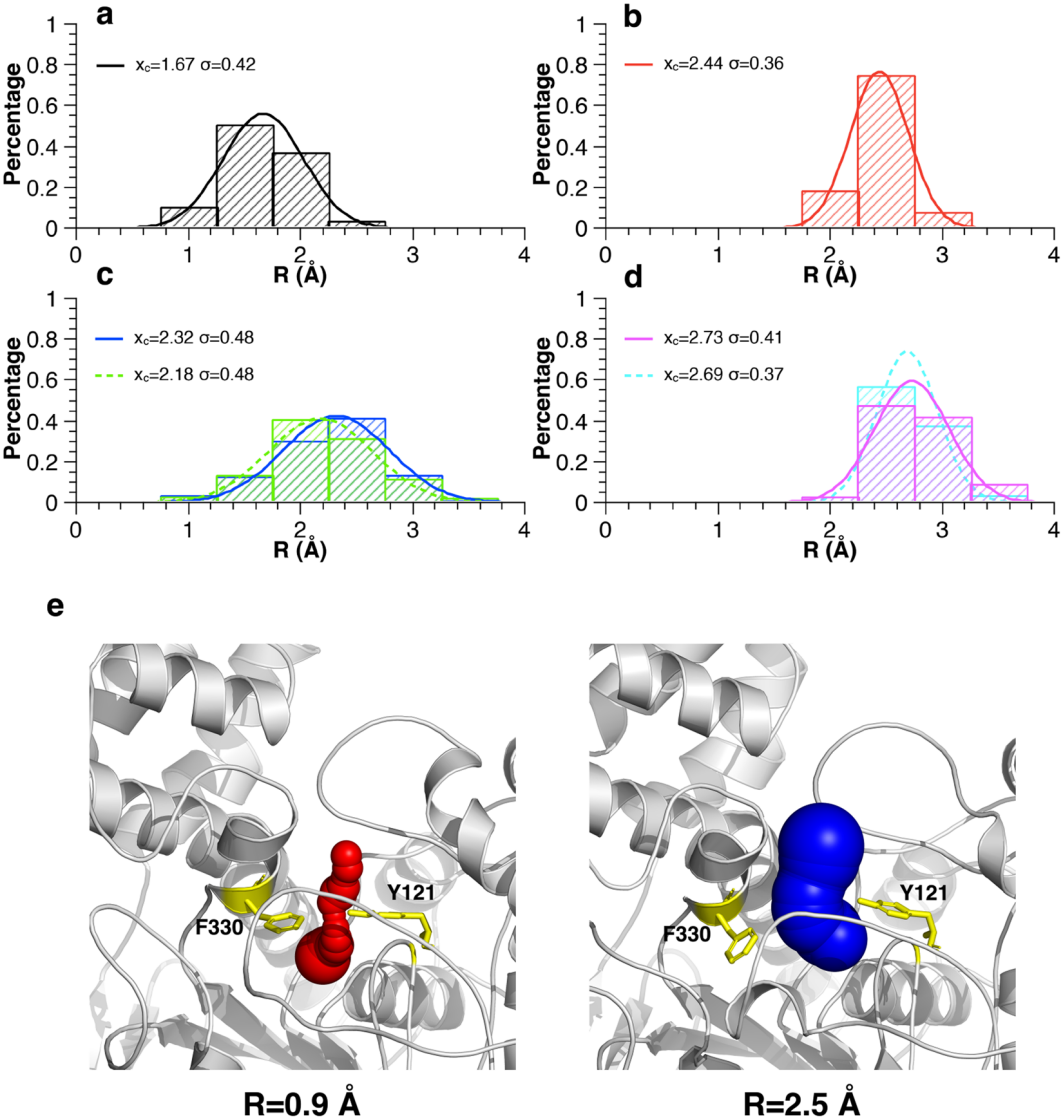
“Breathing” motions of the active-site gorge in simulations revealed by distributions of the minimal gorge radius (R). The histograms of R were normalized by dividing the calculated R values into 8 bins with the width of 0.5 Å and fitted by Gaussian curves ($y={y}_{0}+A\sqrt{2/\pi }{e}^{-\frac{{(x-{x}_{c})}^{2}}{2{\sigma }^{2}}}$, y_0_ = 0 and A=0.5, x_c_ is mean of the distribution and σ is standard deviation) for the monomer *Tc*AChE (**a**), the *Tc*AChE-E2020 complex (**b**), two chains of the apo dimer (**c**), and two chains of the complex dimer (**d**). (**e**) Representative structures of *Tc*AChE extracted at 248 ns and 198 ns with R values of 0.9 and 2.5 Å, respectively, from the trajectory of the monomer *Tc*AChE. Y121 and F330 at the “bottleneck” are shown as yellow sticks.

To monitor the “breathing” motion of the active-site gorge, a simplified way is to calculate the distance between *C*_*ε*2_ of F330 and *O*_*H*_ of Y121 at the “bottleneck” of the gorge^6^. Alternatively, probes with different sizes are placed at the “bottleneck” to detect the open or close gating of the gorge^6, 28^. Here we applied the CAVER software^36, 37^ to determine the cavities inside the enzyme and calculate the radius profile of the long and narrow gorge. Snapshots for the radius calculations were extracted from the trajectories at 1-ns intervals. The size of a probe able to access the “bottleneck”, the narrowest region of the gorge, often corresponds to the minimal radius of the entire gorge. The calculated time-dependent minimal radii (R) are shown in Fig. S3 in the Supporting Information and the distribution of minimal radii are shown in Fig. 2a–d. In the monomer simulation, the minimal radius ranges from 0.9 to 2.52 Å, with an average value of 1.41 ± 0.3 Å and a Gaussian fitted value of 1.67 ± 0.42 Å (Fig. 2a), which is close to previously reported data (1.5 ± 0.26 Å)^6^. The gorge of the monomer *Tc*AChE is found to be shrinking in the simulation, as the average is about 1 Å smaller than that of the starting crystal structure (2.4 Å). Such shrinking is prevented by the bound ligand E2020 or the dimerization of the enzyme; the averaged minimal radius of the gorge increases from 1.41 ± 0.3 Å in the monomer to 2.17 ± 0.19 Å in the complex, 2.0 ± 0.46 Å or 1.96 ± 0.47 Å in two chains of the apo dimer, and 2.52 ± 0.30 Å or 2.46 ± 0.26 Å in the complex dimer. In accord with this, the Gaussian fitted value of R is also gradually increased from the monomer (1.67 ± 0.42 Å) to the complex (2.44 ± 0.36 Å), the apo dimer (2.32 ± 0.48 Å, 2.18 ± 0.48 Å), and the complex dimer (2.73 ± 0.41 Å, 2.69 ± 0.37 Å) (Fig. 2a–d). If a 2.4-Å probe is used to mimic the size of acetylcholine^28^, and therefore defining the gorge as “open” when the radius is greater than 2.4 Å, occupation of the open state of the gorge increases from 0.2% in the monomer to 13.8% in the complex, 20.6% and 17.5% in the two chains of the apo dimer, and 50.5% and 40.6% in the two chains of the complex dimer. Accordingly, ligand binding and dimerization both increase the opening event of the gorge, while the different distribution of R for the complex and the apo dimer (Fig. 2b,c) as well as a P-value of 0.03 in-between these two datasets suggest that mechanisms by which ligand binding or dimerization to have an influence on gorge motions are different. Although they are different, it seems that these two effects are cooperative as the averaged or Gaussian fitted value of R in the complex dimer is the largest among the four simulations. In conclusion, the fluctuations of the minimal radius reveal that the active-site gorge of the enzyme undergoes “breathing” motions in all simulations, while different distributions together with averages of the minimal radius indicate that the pattern of gorge motions varies in the monomer, complex and dimer.

### Influence of residue fluctuations on variations in the gorge radius

**Figure 3:**
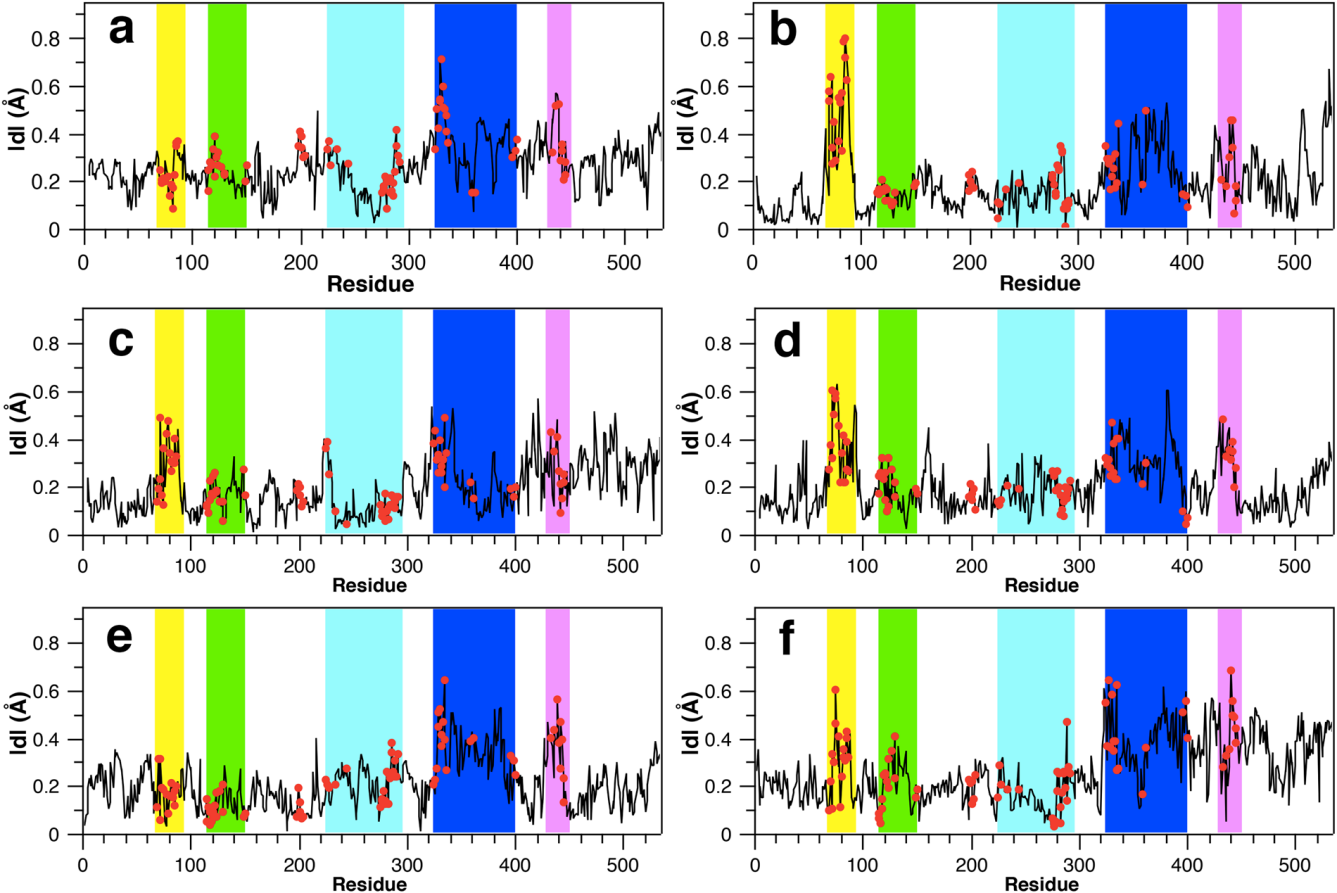
Relevance of residue fluctuations to the minimal radius of the active-site gorge in simulations of the *Tc*AChE monomer (**a**), the *Tc*AChE-E2020 complex (**b**), two chains of the apo dimer (**c,d**), and two chains of the complex dimer **(e,f)**. Gorge residues are shown as red dots. Five subdomains are highlighted in yellow (the Ω-loop), green (S1), cyan (S2), blue (S3), and magentas (S4), the same colors used in Fig. 1a.

To evaluate the residue dynamics in relation to the gorge motions, an absolute value of |*d*_*n*_| is calculated on the basis of atomic coordinates and the minimal radius of the active-site gorge (see Equations 1 and 2 in Method Section) (Fig. 3). The |*d*_*n*_| is a measurement of the deviation along the minimal gorge radius, as distinct from the residue root-mean-square fluctuation (RMSF), which measures deviation from its equilibrium. In addition, to distinguish from other residues, we define any residue within a distance of 10 Å to the bound inhibitor E2020 as a gorge residue, which constructs the inner wall of the active-site gorge. There are 86 gorge residues in total: Q69-F75, F78-P86, W114-S124, L127, V129, Y130, Y148, V150, G198-G203, Q225-G227, W233, R244, I275-S291, N324, D326-A336, L358, P361, V395, N399, V400, W432, M436, and I439-E445. It is observed that most gorge residues can be assigned to five subdomains around the gorge, namely the Ω-loop, subdomains I (S1), II (S2), III (S3), and IV (S4). Their locations with regard to the active-site gorge are shown in Fig. 1a. It looks like that S1 and S4 underpin the gorge, S2 (a three-helix bundle) and S3 (a four-helix bundle) sit on the top of the gorge in an opposite direction, and the Ω-loop just stands by the gorge.

According to the |*d*| values shown in Fig. 3, residues at the C-terminal portion of the enzyme (usually after residue 300), such as in the region of S3 or S4, in general, have larger |*d*| values than those at the N-terminal portion except the Ω-loop, suggesting they are more relevant to the gorge motions. In addition, dynamics of subdomains S3, S4 and the Ω-loop are highly correlated to the variation of the gorge radius, with the exception that in the monomer and one chain of the complex dimer the effect of the Ω-loop is not prominent. In agreement with this, the RMSDs of five subdomains in four simulations reveal that S3, S4 and the Ω-loop are more mobile than S1 and S2 (Figs S1–S2 in Supporting Information). Notably, the |*d*_*n*_| values of residues in the Ω-loop to the radius variation are extremely high in the *Tc*AChE-E2020 complex. The reason for such a remarkable effect is not clear. Another interesting subdomain is S3. In this subdomain, not only the gorge residues but also some of the non-gorge residues exert remarkable effects on the gorge motions (Fig. 3).

**Figure 4:**
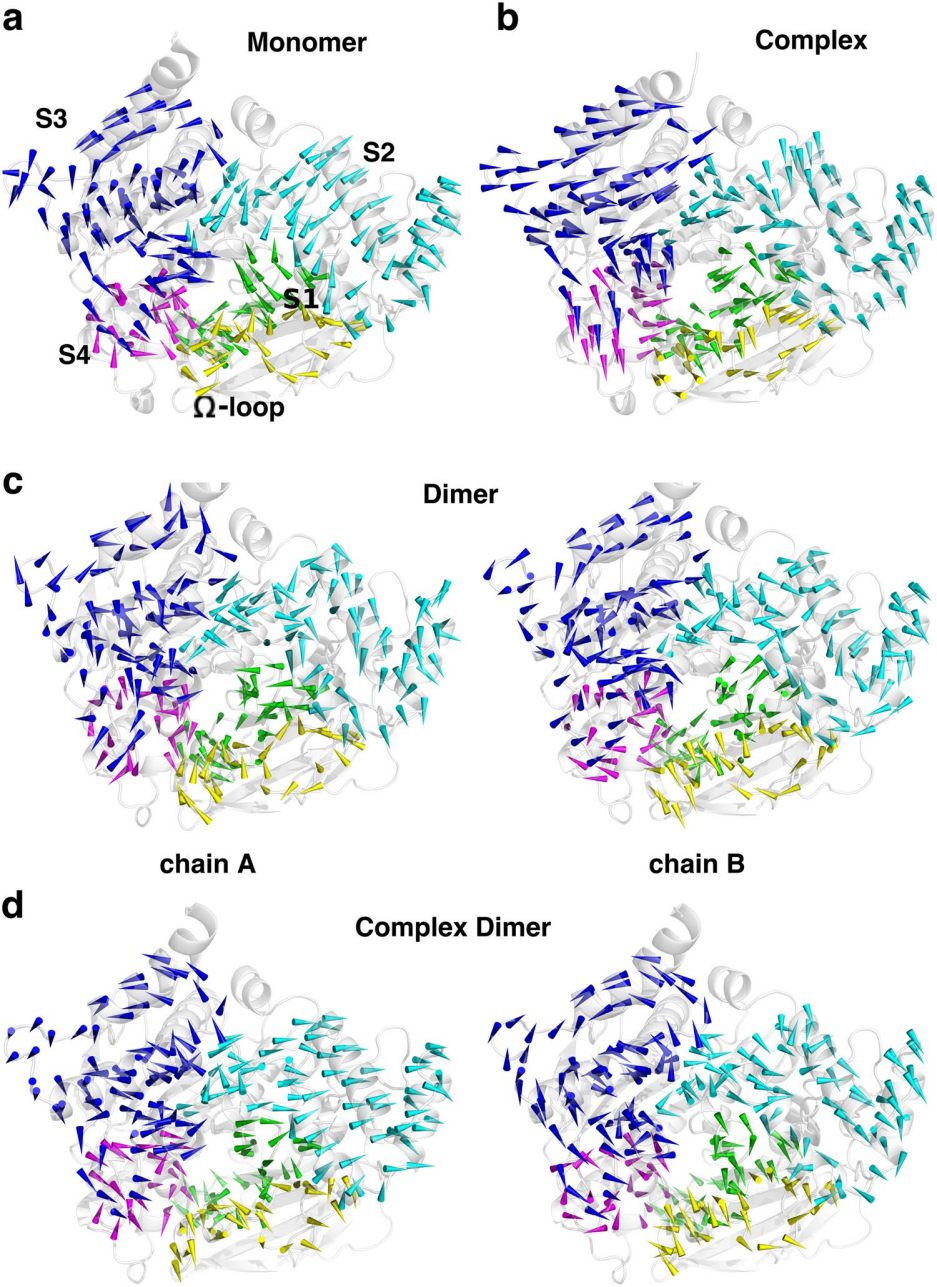
Gorge-radius-relevant movements of residues at five subdomains. Vectors of ***d***_*n*_ mapped onto each residue of five subdomains in simulations of the monomer (**a**), the complex (**b**), the apo dimer (**c**), and the complex dimer (**d**). The enzymes in plots are viewed from the top of the gorge and colors of five subdomains are the same as they are in Fig. 1a: the Ω-loop (yellow), S1 (green), S2 (orange), S3 (blue), and S4 (magenta).

The vectors of |*d*|s mapped onto each residue of five subdomains show that the residues at S3 in the monomer move towards S4 in an anticlockwise direction (Fig. 4a). The squeeze of S3 with S4 drives one segment (residues 325–340) of S3 to move toward the central axis of the gorge and thereby reduces the gorge radius remarkably. Such a movement is not observed for S3 in the simulations of complex and dimer (Fig. 4). In the complex, except the segment including residues 325–340, the rest of the residues at S3 moved toward S2 in a clockwise direction, which is opposite to the monomer (Fig. 4b). The movements of residues at S3 in the dimer are much more anisotropic compared to those in the monomer or complex (Fig. 4c). In the complex dimer, the perturbation from the dimer interface is reduced in the presence of the ligand (Fig. 4d), in company with a reduced RMSD of S3 (Fig. S1c,d). For the Ω-loop, it seems to move towards the axis of the gorge in the monomer and chain A of the dimer but in an opposite way in chain B. Notably, this loop in the complex moves up and down in parallel to the axis of the gorge, resulting in the abnormally large movements observed in the time range ~200–400 ns of the simulation (Fig. S1b). Analogously, the radius-relevant movements of residues at the subdomain S4 are anisotropic (Fig. 4 and Fig. S1). In summary, the highly dynamic subdomains S3, S4 and the Ω-loop play crucial roles in prompting the “breathing” motions of the gorge, while the bound inhibitor or dimerization change the dynamic pattern of these key subdomains.

### Correlated motions of residues

It has been noticed that |*d*| values of gorge residues are not always greater than their neighboring or other non-gorge ones (Fig. 3). Several gorge residues have relatively low |*d*| values (<0.1 Å), which are listed in Table S1. By contrast, some remote residues mainly located at S3 and the C-terminal regions with a high |*d*| value (>0.4 Å) are more relevant to the minimal gorge radius (Table S1). It is thus speculated that not only gorge residues but also distant residues could have an influence on the gorge radius variations. In other words, modulation of the gorge “breathing” motions via allosteric conformational changes occurs with the distant residues during the simulations.

In order to understand how the distant residues exert an allosteric influence on the gorge motions, and how the influences change upon ligand binding or the dimerization on the dynamic network, the program MutInf is used to analyze the correlation of dynamics or conformational changes among residues. MutInf utilizes an entropy-based approach to analyze ensembles of protein conformations resulting from MD simulations and subsequently to identify the correlation between the dynamics of residues at different sites^38, 39^. This method has been successfully applied to study the allosteric networks formation in protein kinases and efficiently identify novel allosteric sites for the activation of 15-lipoxygenase on the arachidonic acid metabolite network^38, 40, 41^. Here we make use of it to study the correlated movements of residues of *Tc*AChE in four simulations (see details in Method).

**Figure 5:**
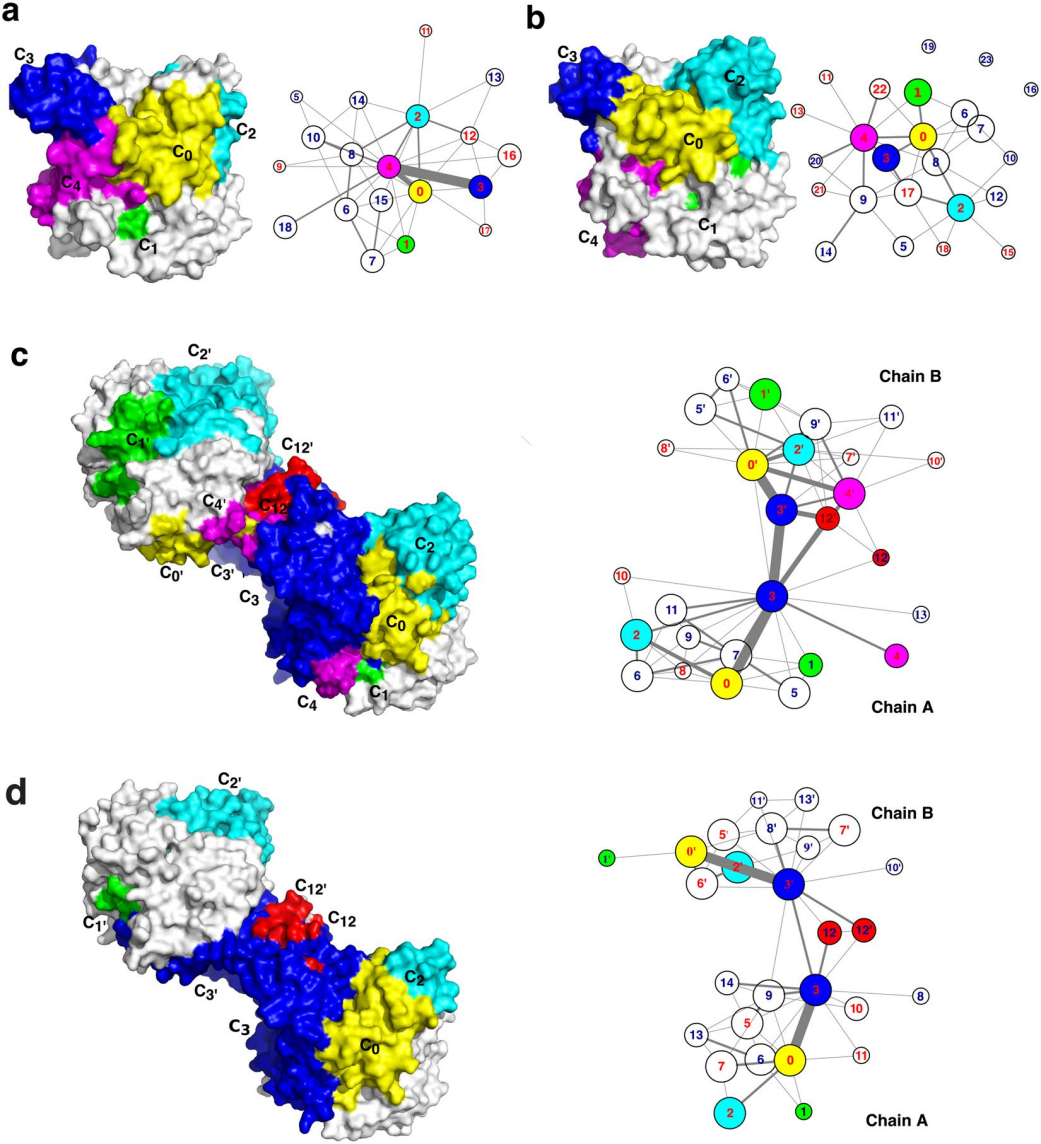
Community maps of *Tc*AChE in four different simulations. (**a–d)** The map of all communities and five communities (C_0_, C_1_, C_2_, C_3_, and C_4_) plotting on the molecular surface of the enzyme for the monomer (**a**), complex (**b**), the apo dimer (**c**), and the complex dimer (**d**), respectively. The number labeled with each node (community) is in accordance with the community number in Table S2 which list all residues included in each community. Communities including gorge residues are labeled with red number. Five communities, C_0_, C_1_, C_2_, C_3_, and C_4_, are colored in yellow, green, cyan, blue, and magenta, respectively. For the dimers, the corresponding communities of the second chain are represented as C_0′_, C_1′_, C_2′_, C_3′_ and C_4′_.

A dynamic community analysis was first performed on each trajectory of four simulations. In such an analysis, MutInf is used to calculate the correlation, in terms of mutual information (*I*), of every two residues with a Cα–Cα distance less than 14 Å that is slightly larger than the cutoff used for the non-bonded electrostatic interactions calculations in the simulations. On the basis of the matrix of *I*, the Girvan-Newman algorithm was then applied to identify the community in which correlated residues often behave collectively. In the resulting graphs shown in Fig. 5, one node represents a community, the size of a node is proportional to the number of residues included in the community, and the thickness of edge weights the total mutual information between every two communities. The number of identified communities from the simulations of monomer, complex, two chains of the apo dimer and of the complex dimer ranges from 17 to 27 (Fig. 5). Residues included in every community are listed in Table S2 in Supporting Information.

Among these communities, C_0_, C_1_, C_2_, C_3,_ and C_4_, have been noticed because most residues involved in these five communities are overlapped with residues in the aforementioned five subdomains which are surrounding the active-site gorge and affect motions of the gorge predominantly (Table S2 and Fig. 5). Importantly, some correlations among these five communities are much stronger than the others (Fig. 5). In the simulation of monomer, C_4_ is the crucial and largest community containing 97 residues. Most residues of S4 and several residues of the Ω-loop, S1, S2 and S3 are included in this community (Table S2). This community is highly correlated with its neighboring communities C_0_ and C_3_, yet its correlation to C_1_ or C_2_ is weak (Fig. 5a). The correlation between C_0_ and C_3_ or C_1_ is weak too. There is no correlation detected between C_3_ and C_1_ or C_2_. In the complex, correlations among the communities are much weaker than those from the monomer. The largest community C_4_ contains 77 residues and it moves correlatively with the community of C_0_ or C_3_, while C_0_ has an equivalent correlation to C_1_, C_2_ and C_3_, respectively. In two chains of the apo dimer, C_3_ is enlarged with more residues from S4, the Ω-loop and S2, and thus becomes the largest community with highest correlations to C_0_ (Fig. 5c). Strikingly, the two communities of two chains, C_3_ and C_3′_, have strong correlations directly or indirectly via C_12′_ which contains the interface residues (Table S2). Accordingly, the correlated motions are speculated to occur among four communities, C_0_-C_3_-C_3′_-C_0′_. The subdomain S3 and the Ω-loop are mainly included in the communities C_3_ and C_0_, respectively. Therefore, if the two S3s move in a seesawing mode, it will affect the movement of two Ω-loops, resulting increased gorge radius in one chain and reduced gorge radius in the other chain simultaneously. This might explain why the size of gorge radius in two chains changed in an opposite way during the 200–400 ns of the trajectory (Fig. S3c). In comparison to the apo dimer, the distinct change in the complex dimer is that the dimer interface interactions in between two C_3_ communities become weak but the correlations between C_3_ and C_0_ inside each chain are still strong.

In summary, though the number of the communities as well as residues included in the communities is varying in four simulations, the correlation between C_3_ and C_4_ is always the strongest while the next one is between C_0_ and C_4_ or C_0_ and C_3_. In addition, the bound inhibitor greatly reduces correlations among the communities whereas the correlation between C_0_ and C_3_ in both chains of the apo dimer is stronger than that in the monomer.

**Figure 6:**
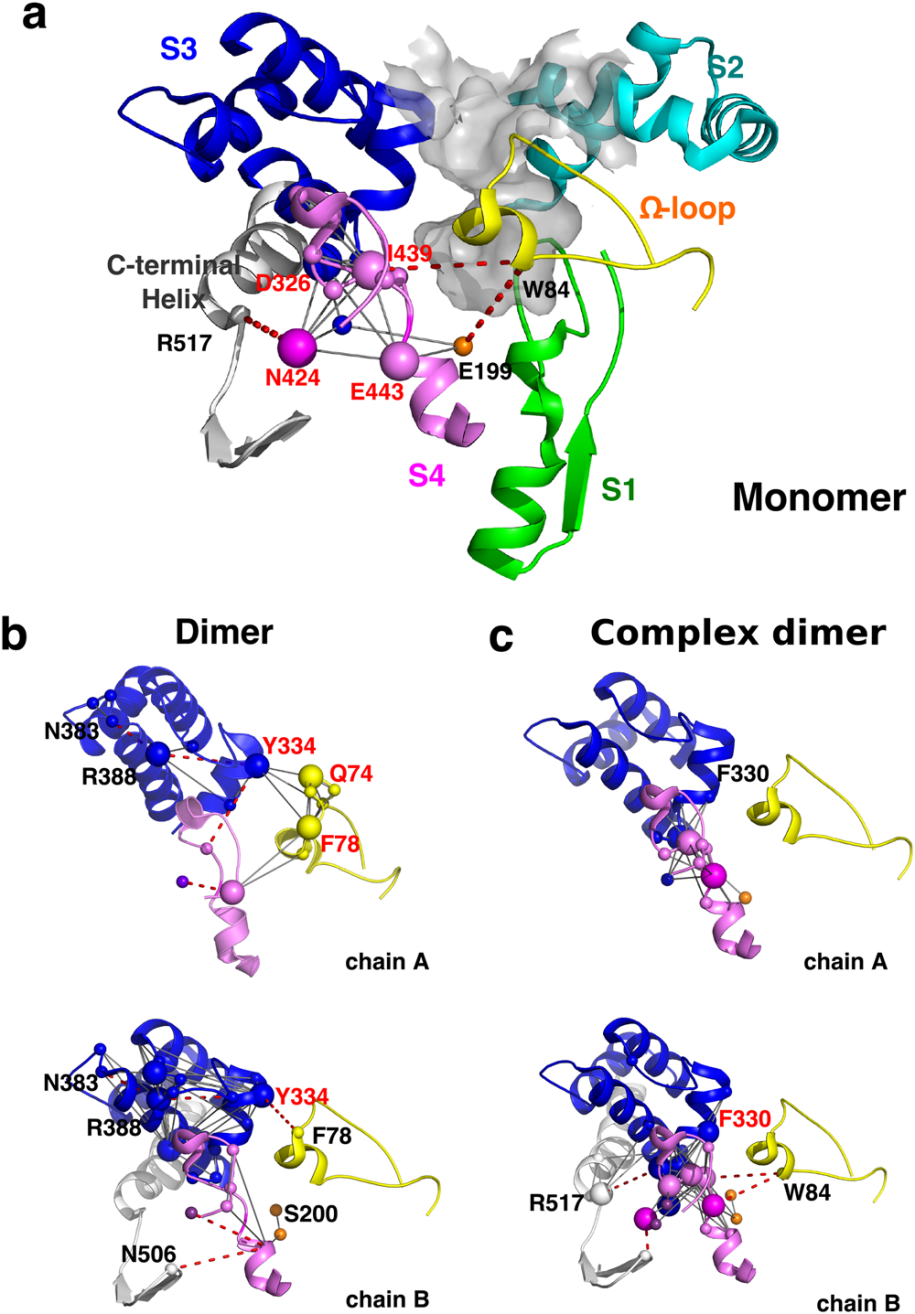
Dynamic communication pathways from distant regions to the gorge in simulations of monomer (**a**), two chains of the apo dimer (**b**) and complex dimer (**c**). Residues of five subdomains are colored according to their belongings. Key residues interacting with multiple residues are shown with large spheres. The probable pathways are plotted as dotted lines.

Besides these five noticed communities, many other communities contain the gorge residues too (Fig. 5 and Table S1). As members of one community, gorge residues move together with non-gorge ones. In this way, the non-gorge residues exert an influence on motions of the gorge too. In an attempt to find out the probable route(s) for the dynamic communication from remote residues to the gorge, each of 86 gorge residues is taken as an initial point to search the most correlated residues (*I* > 1 KT) and then extend to the next layer until the route is ended. Again, a Cα–Cα distance of 14 Å is taken as a cutoff to pick up residues for the correlation (*I*) calculations. For the complex, it fails to find such a pathway due to the weak correlations in-between the communities or residues (Fig. 5b). For the monomer and dimer, there are several routes observed (Fig. 6). The short routes made up of less than five residues in tandem such as the one including the “bottleneck” residue, G119-F120-Y121-S122, are ignored in order to focus on the non-local residue correlations. The most probable route in the monomer starts from W84, passes through E199 or I439, an interacting interface between S3 and S4 (mainly composed of D326, I439, N424, and E443), and ends with R517 (Fig. 6a). In the apo dimer (Fig. 6b), the communication interface between S3 and S4 becomes incompact, and S3 directly communicates with the Ω-loop and thus transfers the effects from the dimer interface to the gorge wall. A similar route, N383-R388-Y334-F78, is observed for both chains of the dimer. An additional route found in chain B is from N506 at the C-terminal to residues at S4 and then reaches the catalytic residue S200 at the active site (Fig. 6b). By contrast, in the complex dimer, the interface between S3 and S4 becomes more compact again and the “bottleneck” residue F330 is also involved in this interface (Fig. 6c). It is known that the conformational changes of F330 are crucial to the minimal gorge radius. Such a strong linkage of S4 to S3 (including F330), together with the restriction from the bound inhibitor, leads to the greatest gorge radius resulted from the simulation of the complex dimer (Fig. 2). The strong linkage may give an extra pulling force that makes the gorge more open upon the ligand binding of the dimer. In summary, the dynamic communication routes always cross over the subdomains of the Ω-loop, S3 and S4. In other words, dynamics of residues located at S3 or S4 could be transferred to the residues at the Ω-loop. It is consistent with the result from the community analysis that the correlation between C_0_ and C_3_ in the dimer becomes stronger in comparison with the monomer.

## Conclusions

Numerous simulation studies have been performed on AChE to explore function-relevant dynamic structures, thereby enhancing our understanding of its modes of action and providing clues for structure-based drug design targeting the enzyme. Here, we extend simulations of the enzyme from nanosecond to microsecond, from the apo form to the inhibitor-bound complex, and from the monomer to the dimer, so as to comparatively elucidate the intrinsic motions of the enzyme as well as the effects to modulate the motions. The “breathing” motions of the active-site gorge, where the substrate is catalyzed to degradation and inhibitors are bound, are examined by calculation of the minimal gorge radius. Subsequently, contributions of residues, particularly the gorge residues, and five subdomains surrounding the gorge to radius variations are evaluated. To our surprise, not only the gorges residues, but also some distant residues are tightly associated with changes of the radius. Residue-residue correlated dynamics based on the concept of mutual information are thereby analyzed and dynamic communication pathways from remote residues to the gorge are revealed.

Distributions of the minimal radius of the active-site gorge shown in Fig. 2 suggest that “breathing” motions occur with *Tc*AChE in a monomer, complex or dimer form. Such motions are not only caused by the movement of gorge residues but also the surrounding subdomains. Three of the five subdomains, S3, S4 and the Ω-loop, whose dynamics are highly correlated (Fig. 5–6), are mostly relevant to the variation of the gorge radius during the simulations (Fig. 3). In other words, dynamics of these three subdomains exert significant influence on the “breathing” motions of the gorge. In agreement with this, these subdomains are indeed mobile in the simulations (Figs S1–S2). Gorge residues located at these three subdomains are directly involved into the gorge motions while those remote ones could allosterically modulate the gorge motions via the dynamic communication pathways which cross over S3, S4 and the Ω-loop (Fig. 6). It is thus concluded that subdomains S3, S4 and the Ω-loop move in concert with each other to play a predominant role in the “breathing” motion of the active-site gorge.

However, it is apparent that motions of the enzyme differ in four simulations, which is clearly revealed by different distributions of the minimal gorge radius shown in Fig. 2. In comparison to the monomer, the bound inhibitor and dimerization not only increase the gorge radius as well as the opening rate of the gorge but also change the dynamic patterns of the residues as well as subdomains, which, to the best of our knowledge, has been revealed for the first time. The bound E2020 prevents the active-site gorge from shrinking but keeps it in a relatively large and stable form, in particularly in the complex dimer, the minimal gorge radius reaches the largest one among four simulations. It is also the bound E2020 that weakens or even breaks the dynamic correlations among residues and subdomains (Fig. 5). The weak correlation of the Ω-loop to both S3 and S4, which is only observed in the complex, promotes the extremely high movements of this loop. Such weak correlations may also account for changes of the dynamic pattern of residues at five subdomains in comparison to the monomer (Fig. 4). If it is logical to accept that the binding of a ligand in the gorge could modulate the enzyme’s motions, the significant influence of dimerization on motions of the active-site gorge is intriguing at the beginning, as the dimerization interface is far away from the gorge. Figure 2 shows that the dynamic residues at the C-terminal portion of the enzyme contribute more to the gorge radius variations than the N-terminal residues. The C-terminal residues either directly participate into the dimerization or are nearby the monomer-monomer interaction interface. For example, S3 and S4, both from the C-terminal portion, are located in and close to the dimer interface, respectively. Figure 5c suggests that the allosteric modulation of one chain to the other could be achieved through subdomains S3 (with or without S4) and the Ω-loop. ﻿Accordingly, it is proposed that the dimerization effect on the gorge motions could be achieved by modifying the dynamic pattern of S3 first and subsequently affecting the dynamics of S4 or/and the Ω-loop which both have high correlations to S3.﻿

## Methods

### Simulation Systems

Two crystal structures with PDB codes 1ea5 and 1eve were used as the starting structures for the simulations of monomer *Tc*AChE and *Tc*AChE-E2020 complex, respectively. The dimer was generated by the program PyMOL (www.pymol.org) by applying a symmetry operation on the monomer copy. The monomeric *Tc*AChE or the *Tc*AChE-E2020 complex was inserted into a box with a dimensions of 121 × 121 × 121 Å^3^, while a larger box of 177 × 177 × 177 Å^3^ was used for the dimer of *Tc*AChE. The minimal distance between the protein (or the protein-ligand complex) and the box boundary was 20 Å. The boxes were solvated by equilibrated TIP3P water molecules. Ions were added to neutralize the system and to mimic a salt concentration of 0.15 M. Stepwise energy minimizations were performed with position restrictions on heavy atoms, main-chain atoms, Cα atoms, and no atoms, using steepest descent method. Then 300 ps equilibration MD runs were performed, in which the temperature was gradually increased through the range 50 K, 100 K, 150 K, 200 K, 250 K, and 300 K with 50 ps each.

### MD Simulations

MD simulations were carried out with GROMACS software^42^, using NPT and periodic boundary conditions. The CHARMM force field was applied to proteins and the CHARMM general force filed (CGenFF) was used for the inhibitor E2020^43^. The final temperature for three simulations was kept constant at *T *= 300 K by coupling to a velocity rescaling thermostat^44^ with a coupling time of *t*_T_ = 0.1 ps. The pressure was kept constant at *p* = 1 bar using an isotropic coupling to a Berendsen barostat^45^ with a coupling time of *t*_p_ = 0.1 ps and an isotropic compressibility of 4.5 × 10^−5^ bar^−1^. Lennard-Jones interactions were calculated using a cutoff of 14 Å. At distances less than 12 Å, the electrostatic interactions were calculated explicitly, whereas long-range electrostatic interactions were calculated using particle-mesh Ewald summation^46, 47^. Bond lengths between the hydrogen atoms and heavy atoms were constrained using the LINCS algorithm^48^ with an integration time step of 2 fs. The coordinates of the entire system were saved every 5000 steps for the 1 *μ*s simulation of monomeric *Tc*AChE, 1 *μ*s simulation of *Tc*AChE-E2020 complex, 524 ns simulation of *Tc*AChE dimer, and five 100 ns simulations of *Tc*AchE-E2020 dimer. All of them were used for the analysis.

### Cavity Measurement

Snapshots were taken at 1-ns intervals along the trajectories, generating a total number of 1000, 1000﻿, 524﻿﻿,﻿ and 500 snapshots of the *Tc*AChE monomer, the *Tc*AChE-E2020 complex, the apo dimer, and the complex dimer, respectively, for the cavity analysis. CAVER 3.0 was used to measure the size of the cavity inside *Tc*AChE^36, 37^. First, the starting point for the tunnel analysis was determined by probing the cavities inside the protein. Then, the largest probe accessing the deepest site in the pocket detected the tunnel. The inner probe and the outer probe at the protein surface were set as 1.4 Å and 3.0 Å, respectively. The initial radius for the cavity was set as 0.9 Å. The clustering threshold was set a value of 4.5. The seed was set to 1 to ensure consistent results. The shell radius was set to 5.0 Å and the shell depth was set to 10 Å. Other parameters were taken from their default values as listed in the CAVER user guide version 3.0.

### Correlation between Residue Movement and Gorge Radius

To measure the contribution of each residue on the breathing motion of the active-site gorge, the correlation vector of each atom *i* can be calculated by the equation defined by Shen *et al*.^6^:

1 $${{\boldsymbol{\rho }}}_{{\boldsymbol{i}}}=\frac{\langle({{\boldsymbol{r}}}_{{\boldsymbol{i}}}(t)-\langle{{\boldsymbol{r}}}_{{\boldsymbol{i}}}{\rangle}_{t}){(R(t)-{\langle R\rangle}_{t})}_{t}\rangle}{\sqrt{{\langle{({{\boldsymbol{r}}}_{{\boldsymbol{i}}}(t)-{\langle{{\boldsymbol{r}}}_{{\boldsymbol{i}}}\rangle}_{t})}^{2}\rangle}_{t}{\langle{(R(t)-{\langle R\rangle}_{t})}^{2}\rangle}_{t}}}\,$$

where *r* is the Cartesian coordinate of each atom *i *at time *t* and *R* is the radius of the gorge at the same time. The contribution of each residue *n* to the change of the gorge radius can be mass-weighted evaluated by the equation:

2 $${{\boldsymbol{d}}}_{n}=\frac{{\sum }_{i\in n}{m}_{i}{{\boldsymbol{\rho }}}_{i}}{{\sum }_{i\in n}{m}_{i}}$$

### MutInf Method

McClendon *et al*.^38, 39^ developed a method, MutInf, to analyze the MD trajectories to identify statistically significant correlated motions of residues by calculating parameters related to residue-by-residue conformational entropies. It has been illustrated that the pattern of mutual information between residues can be used to identify couplings between allosteric sites and to identify residues that might be important in mediating these couplings^38, 39^.

Using the program MutInf, the 1 *µ*s MD trajectory was split into five “blocks”, each of 200 ns. 40,000 snapshots at an interval of 50 ps were generated for each block. Subsequently, the conformational space of a molecule was described using an internal coordinate system. Only the torsion angles of the *ϕ*, *ψ* of the main-chain and the *χ* torsion angles of the side-chain (only the *χ*_1_ of proline) were used to calculate the entropy and mutual information, ***I***, which was the difference between the self- and the joint entropy, so *I* = *S*(1) + *S*(2) − *S*(1, 2). *S*(1) and *S*(2) were the self-entropy of residues 1 and 2, respectively. *S*(1, 2) was the joint entropy between them. The changes in bond lengths, bond angles and ω backbone torsion angle﻿s﻿﻿ were neglected. Communities were identified by Girvan-Newman algorithm^49^ based on the *I* values. For the dimers, the community analysis is performed on the two chains together.

## Electronic supplementary material

Supplementary Information

## References

[1] Rosenberry TL (1975). Acetylcholinesterase. Adv. Enzymol. Relat. Areas. Mol. Biol..

[2] Silman I, Sussman JL (2005). Acetylcholinesterase: ‘classical’ and ‘non-classical’ functions and pharmacology. Curr. Opin. Pharmacol..

[3] Sussman JL (1991). Atomic structure of acetylcholinesterase from Torpedo californica: a prototypic acetylcholine-binding protein. Science.

[4] Kryger G, Silman I, Sussman JL (1999). Structure of acetylcholinesterase complexed with E2020 (Aricept): implications for the design of new anti-Alzheimer drugs. Structure.

[5] Colletier JP (2006). Structural insights into substrate traffic and inhibition in acetylcholinesterase. EMBO J..

[6] Shen T, Tai K, Henchman RH, McCammon JA (2002). Molecular dynamics of acetylcholinesterase. Acc. Chem. Res..

[7] Brady R, Weinman J (2013). Adherence to cholinesterase inhibitors in Alzheimer’s disease: a review. Dement. Geriatr. Cogn. Disord..

[8] Anand P, Singh B (2013). A review on cholinesterase inhibitors for Alzheimer’s disease. Arch. Pharm. Res..

[9] Bajda M (2013). Structure-based search for new inhibitors of cholinesterases. Int. J. Mol. Sci..

[10] Quinn DM (1987). Acetylcholinesterase: Enzyme Structure, Reaction Dynamics, and Virtual Transition States. Chem. Rev..

[11] Taylor P, Radic Z (1994). The cholinesterases: from genes to proteins. Annu. Rev. Pharmacol. Toxicol..

[12] Kingsley LJ, Lill MA (2015). Substrate tunnels in enzymes: structure-function relationships and computational methodology. Proteins.

[13] Fang L, Pan Y, Muzyka JL, Zhan CG (2011). Active Site Gating and Substrate Specificity of Butyrylcholinesterase and Acetylcholinesterase: Insights from Molecular Dynamics Simulations. J. Phys. Chem. B.

[14] Gilson, M. K. *et al*. Open “back door” in a molecular dynamics simulation of acetylcholinesterase. *Science* **263**, 1276–1278 (1994). 10.1126/science.8122110 8122110

[15] Bui JM, Tai K, McCammon JA (2004). Acetylcholinesterase: Enhanced Fluctuations and Alternative Routes to the Active Site in the Complex with Fasciculin-2. J. Am. Chem. Soc..

[16] Wlodek ST, Clark TW, Scott LR, McCammon JA (1997). Molecular Dynamics of Acetylcholinesterase Dimer Complexed with Tacrine. J. Am. Chem. Soc..

[17] Van Belle D, De Maria L, Iurcu G, Wodak SJ (2000). Pathways of Ligand Clearance in Acetylcholinesterase by Multiple Copy Sampling. J. Mol. Biol..

[18] Xu Y (2010). Long route or shortcut? A molecular dynamics study of traffic of thiocholine within the active-site gorge of acetylcholinesterase. Biophys. J..

[19] Bennion BJ (2015). A wrench in the works of human acetylcholinesterase: soman induced conformational changes revealed by molecular dynamics simulations. PLoS. One.

[20] Xu Y (2003). How does huperzine A enter and leave the binding gorge of acetylcholinesterase? Steered molecular dynamics simulations. J. Am. Chem. Soc..

[21] Niu C (2005). Dynamic Mechanism of E2020 Binding to Acetylcholinesterase: A Steered Molecular Dynamics Simulation. J. Phys. Chem. B.

[22] Tai K, Shen T, Borjesson U, Philippopoulos M, McCammon JA (2001). Analysis of a 10-ns molecular dynamics simulation of mouse acetylcholinesterase. Biophys. J..

[23] Antosiewicz J, McCammon JA, Wlodek ST, Gilson MK (1995). Simulation of charge-mutant acetylcholinesterases. Biochemistry.

[24] Antosiewicz J, Wlodek ST, McCammon JA (1996). Acetylcholinesterase: role of the enzyme’s charge distribution in steering charged ligands toward the active site. Biopolymers.

[25] Tan RC, Truong TN, McCammon JA, Sussman JL (1993). Acetylcholinesterase: electrostatic steering increases the rate of ligand binding. Biochemistry.

[26] Tara S (1998). Rapid binding of a cationic active site inhibitor to wild type and mutant mouse acetylcholinesterase: Brownian dynamics simulation including diffusion in the active site gorge. Biopolymers.

[27] Szabo A, Shoup D, Northrup SH, McCammon JA (1982). Stochastically gated diffusion-influenced reactions. J. Chem. Phys..

[28] Zhou HX, Wlodek ST, McCammon JA (1998). Conformation gating as a mechanism for enzyme specificity. Proc. Natl. Acad. Sci. USA.

[29] Xu Y (2008). Induced-fit or preexisting equilibrium dynamics? Lessons from protein crystallography and MD simulations on acetylcholinesterase and implications for structure-based drug design. Protein Sci..

[30] Xu Y (2008). Flexibility of aromatic residues in the active-site gorge of acetylcholinesterase: X-ray versus molecular dynamics. Biophys. J..

[31] Lee Y, Jong LS, Choi S, Hyeon C (2011). Link between allosteric signal transduction and functional dynamics in a multisubunit enzyme: S-adenosylhomocysteine hydrolase. J. Am. Chem. Soc..

[32] Lee Y, Choi S, Hyeon C (2014). Mapping the intramolecular signal transduction of G-protein coupled receptors. Proteins.

[33] Lee Y, Choi S, Hyeon C (2015). Communication over the network of binary switches regulates the activation of A2A adenosine receptor. PLoS. Comput. Biol..

[34] Lee Y, Kim S, Choi S, Hyeon C (2016). Ultraslow Water-Mediated Transmembrane Interactions Regulate the Activation of A2A Adenosine Receptor. Biophys. J..

[35] Sussman JL (1988). Purification and crystallization of a dimeric form of acetylcholinesterase from Torpedo californica subsequent to solubilization with phosphatidylinositol-specific phospholipase C. J. Mol. Biol..

[36] Chovancova E (2012). CAVER 3.0: a tool for the analysis of transport pathways in dynamic protein structures. PLoS. Comput. Biol..

[37] Kozlikova B (2014). CAVER Analyst 1.0: graphic tool for interactive visualization and analysis of tunnels and channels in protein structures. Bioinformatics.

[38] McClendon CL, Kornev AP, Gilson MK, Taylor SS (2014). Dynamic architecture of a protein kinase. Proc. Natl. Acad. Sci. USA.

[39] McClendon CL, Friedland G, Mobley DL, Amirkhani H, Jacobson MP (2009). Quantifying Correlations Between Allosteric Sites in Thermodynamic Ensembles. J. Chem. Theory. Comput..

[40] Srivastava AK (2014). Synchronous opening and closing motions are essential for cAMP-dependent protein kinase A signaling. Structure.

[41] Meng H (2016). Discovery of Novel 15-Lipoxygenase Activators To Shift the Human Arachidonic Acid Metabolic Network toward Inflammation Resolution. J. Med. Chem..

[42] Pronk S (2013). GROMACS 4.5: a high-throughput and highly parallel open source molecular simulation toolkit. Bioinformatics.

[43] Vanommeslaeghe K (2010). CHARMM general force field: A force field for drug-like molecules compatible with the CHARMM all-atom additive biological force fields. J. Comput. Chem..

[44] Bussi G, Donadio D, Parrinello M (2007). Canonical sampling through velocity rescaling. J. Chem. Phys..

[45] Berendsen HJC, Postma JPM, van Gunsteren WF, DiNola A, Hakk JR (1984). Molecular dynamics with coupling to an external bath. J. Chem. Phys..

[46] Darden T, York D, Pedersen L (1993). Particle mesh Ewald: An N-log(N) method for Ewald sums in large systems. J. Chem. Phys..

[47] Essmann U (1995). A smooth particle mesh ewald potential. J. Chem. Phys..

[48] Hess B, Bekker H, Berendsen HJC, Fraaije JGEM (1997). LINCS: A linear constraint solver for molecular simulations. J. Comput. Chem..

[49] Girvan M, Newman MEJ (2002). Community structure in social and biological networks. Proc. Natl. Acad. Sci. USA.

[50] Wallace AC, Laskowski RA, Thornton JM (1995). LIGPLOT: a program to generate schematic diagrams of protein-ligand interactions. Protein Eng..

