# Supplementary material for: Gorge Motions of Acetylcholinesterase Revealed by Microsecond Molecular Dynamics Simulations
Source: Sci Rep. 2017 Jun 12;7:3219. doi: 10.1038/s41598-017-03088-y (PMC5468367; doi:10.1038/s41598-017-03088-y)
Supplement: Supplementary file 1 — Supplementary Information [file 41598_2017_3088_MOESM1_ESM.pdf]

Supporting Information

Gorge Motions of Acetylcholinesterase Revealed by Microsecond Molecular Dynamics Simulations

Shanmei Cheng, Wanling Song, Xiaojing Yuan, Yechun Xu*

CAS Key Laboratory of Receptor Research, Drug Discovery and Design Center, Shanghai Institute of Materia Medica, Chinese Academy of Sciences (CAS), Shanghai 201203, China.

Table of Contents:

Figure S1·····Page S2

Figure S2····· Page S3

Figure S3····· Page S4

Table S1·····Page S5

Table S2····· Page S6-10

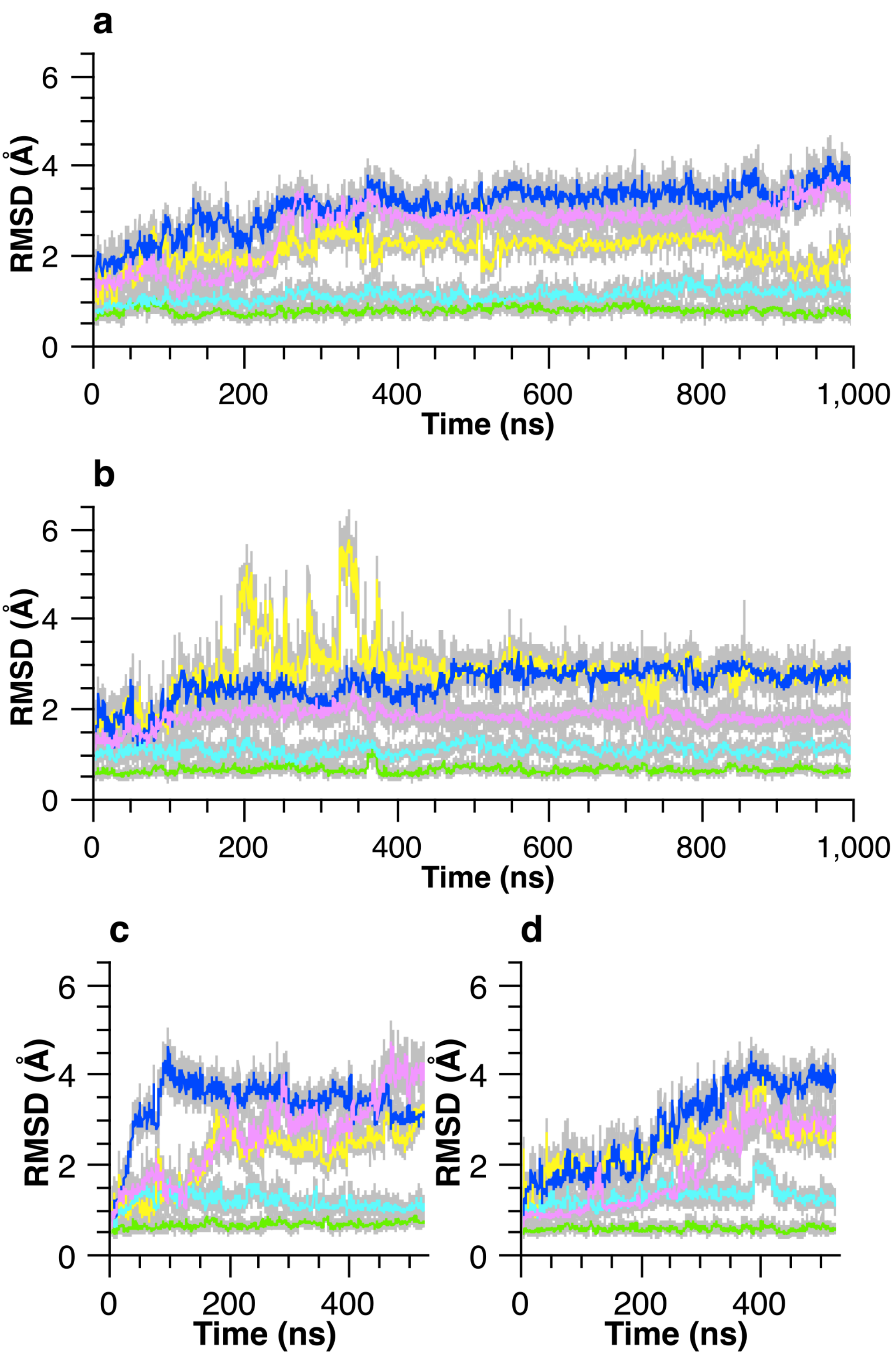

Figure S1. Movements of five subdomains surrounding the active-site gorge. Backbone RMSDs of the Ω-Loop (yellow), S1 (green), S2 (cyan), S3 (blue), and S4 (magenta) versus time in simulations of the monomer (**a**), complex (**b**) and apo dimer (**c**, **d**).

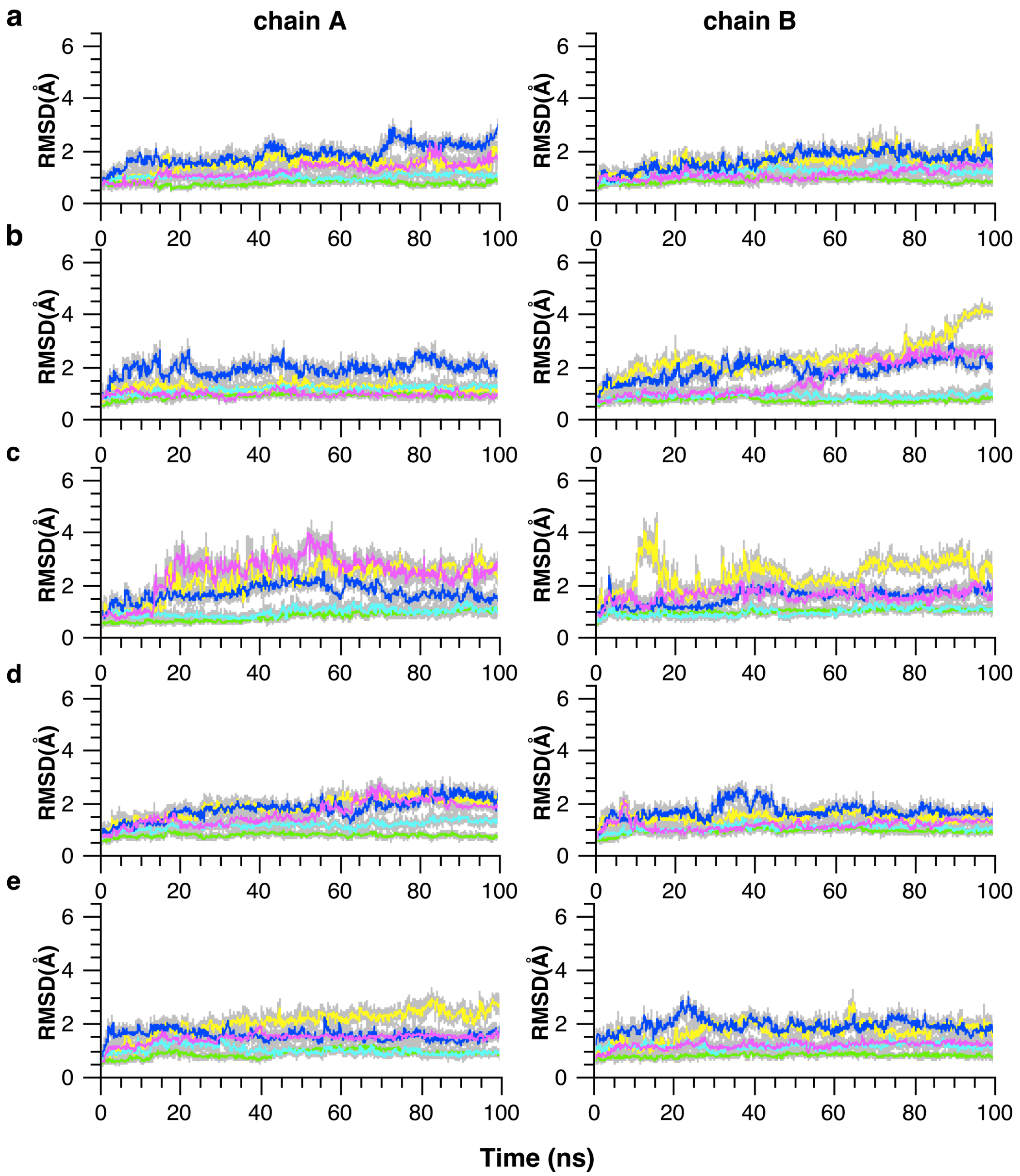

Figure S2. Movements of five subdomains surrounding the active-site gorge in five 100-ns simulations of *Tc*AChE dimer in complex with E2020. (a-e) Backbone RMSDs of the Ω-Loop (yellow), S1 (green), S2 (cyan), S3 (blue), and S4 (magenta) versus time of the simulations.

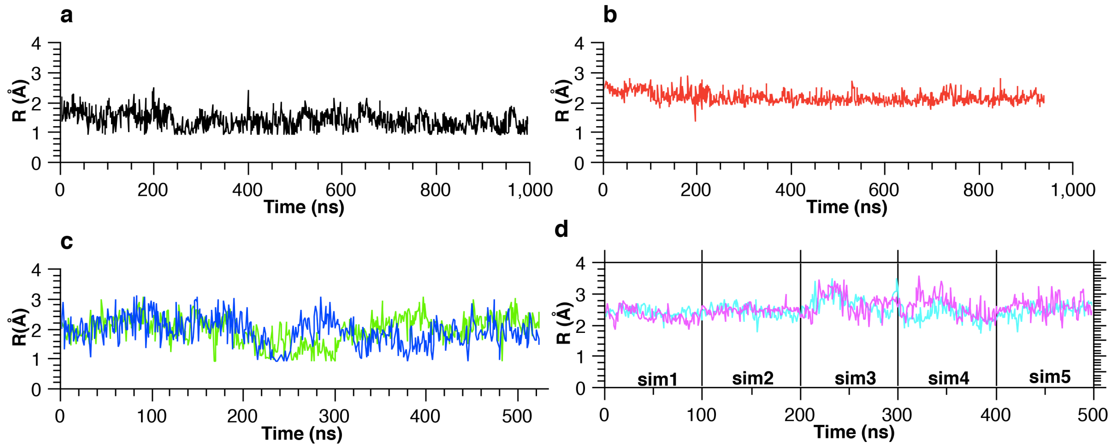

Figure S3. Time-dependent minimal gorge radius (R) in simulations of the *Tc*AChE monomer (**a**), the *Tc*AChE-E2020 Complex (**b**), the apo dimer (**c**), and the complex dimer (**d**).

Table S1 Gorge residues with low |d| values and non-gorge residues with high |d| values.

	Gorge Residues (|d|<0.1Å)	Non-gorge residues (|d|>0.4 Å)
**Monomer**	E82, N280	R216, L321, G322, N364-V371, T376, G390-D393, L420, Y421, A427, P433-W435, G437, V438, F523, W524, F527-A534
**Complex**	S226, S286, I287, R289, V400, E443	N65, N87, I347-R349, D351, N364-L366, L368, D369, Y375, D380, D381, N424, H425, V438, D504-T507, W524-N533
**Chain A of the Dimer**	Y116, Y130, R244, V277, E278, N280-L282, G441	R88, I223, G322, V323, S340-S343, N416, Y421, V438, W473, A474, P482, N483, N488, E489, W492, P493, F523, A534
**Chain B of the Dimer**	L282, S286, N399, V400	R47, P76, G77, E92, D93, G160, P337-K341, S343, E344, K346, D380-I385, N387, N429, L430, G437, V438
**Chain A of the Complex Dimer**	V71, S79, I115, G117-Y121, S124, Y130, Y148, V150, G198, A201-G203	R216, S340-S345, R349, S359, V360, L373, Q374, D380, D381, I385, K386, R426, L430, V438
**Chain B of the Complex Dimer**	W114-Y116, I275-E278, P283	R88, G322, V323, K341, S345, D365, L366, D369, A370, T372-D381, N383, I385, K386, R388, G390, D392, D393, G396-H398, I401, 410, N416, L420-H425, A427-L430, F446, P451, L452, Y458-A460, E463-I469, H471, W473, F502, I503, L505-T507, V512, R517, Q519, M520, V522, F523, Q526-T535

Table S2. Residues included in communities resulted from the dynamic correlation analysis of the monomer (I), the complex (II), two chains of the apo (III-IV) and complex (V-VI) dimer. Residues located at five subdomains are labeled with the same color as those shown in Fig. 1.

I

#Community	Residues	Subdomains
C_0	N65, N66, C67-Q74, P76, G77, G80-M83, N85-C94, L95-N98, W114-L127， S145-S147, K269-W279	Ω-loop+S1+S2
C_1	Y130-V144	S1
C_2	P38-E49, Y148-V150, G151-V168, V236-G249, D259, I263-E268, V293-I296, D297-E299, F301	S1+S2
C_3	L333-M353, S359, D365, G367-R388, L391, D392， I394, V395, W435, Q519-M520	S3+S4
C_4	F75, F78, S79, W84, D128, V129, F197-S200, L224, N225-C231, L320-V323, N324-L332, D389, D393, I401-V408, L420-A427, S428-E434， M436-F446, E461, R468-W473, K491, W492, K498-V518	Ω-loop+S1+S2+S3+S4
C_5	E484-S490	/
C_6	S108-V113, N183-I196, A474-G480	/
C_7	S4-V8, V15-F30, N59-P64, I99-K107	/
C_8	G169-D182, G207-R220, F300, N313-K315	/
C_9	A201-V206	/
C_{10}	R221-I223, K316-I319, N409-Y419, N481-N483, P493-T497	/
C_{11}	P232-S235	S2
C_{12}	R250, N251, N280-F292	S2
C_{13}	C254-S258, E260-L262	S2
C_{14}	S304-G312	/
C_{15}	N9-K14, L31-E37, P50-W58	/
C_{16}	S354-L366, C521-A534	S3
C_{17}	G396-V400	S3
C_{18}	V447-L450, P451-A460, E462-R467	S4

II

#Community	Residues	Subdomains
C_0	N65, N66, C67-M90, G119-D128, Y130, S147, I275, S329-A336, K341	Ω-loop+S1+S2+S3
C_1	L95-V101, L135-V150, G151	S1
C_2	E37-R46, S91-C94, A152-A164, V238, E240-L274, D276-L282, V295	Ω-loop+ S2
C_3	L332, P337-S340, D342-F352, L373-D392, L394-D397, E434-G437	S3+S4
C_4	W114, V129, F197-S200, Q225-G227, L320-V323, N324-G328, D393, L404, Y419-A427, V438-L450, P451, L452, R467-W473, N483-P493, I503-H513, R517, Q519-C521	S1+S2+S3+S4
C_5	R47-K52, I296, D297-S304, E306-M308	S2
C_6	D2-L6, R19-S28, P102-R105, N131-Y134	S1
C_7	L7-T18, P53-P64	/
C_8	P106-V111, D182-I196	/
C_9	M112, V113, R221-L224, S228-C231, L309-I319, M405-T418, A474-P482, L494-T497	S2
C_{10}	A29-A36	/
C_{11}	I115-G118	S1
C_{12}	P165-H181	/
C_{13}	A201-S205	/
C_{14}	V206-H209, T210-R220	/
C_{15}	P232-S237	S2
C_{16}	A239	S2
C_{17}	P283-I287, M353-T372, V522-A534	S2+S3
C_{18}	F288-P294	S2
C_{19}	L305	/
C_{20}	K498-F502, Q514-V518	/
C_{21}	H398-V400, I401-P403	S3
C_{22}	S428-W432, V453-S466	S4
C_{23}	P433	S4

III

#Community	Residues	Subdomains
C_0	Chain A: T62-N66, C67, Q69-E73, E82, N85-M90, D93, Y96-N98, W114, Y121-D128, S145-S147, Q272, E278, W279	Chain A: Ω-loop+S1+S2
C_1	Chain A: I99, W100, K133-V144	Chain A: S1
C_2	Chain A: P38-R46, Q68, S91, E92, C94, L95, G119, F120, Y148-V150, G151-V168, V236, R242-V277, N280-P283, S291, F292, I296	Chain A: Ω-loop+S1+S2
C_3	Chain A: Q74-S81, M83, W84, I115-G118, V129-G132, F197-S200, L224, Q225-N230, F284-F290, L320-V323, N324-M353, K357-D369, Q374-D377, N382-V400, I401-F407, L420-A427, S428-F448, Y458-E462, R468-W473, N483-P493, K498-V522, W524-P529, L531, L532, A534;<br>Chain B: D365, L373, M379, D380, K530	Chain A: Ω-loop+S1+S2+S3+S4<br><br>Chain B: S3
C_4	Chain A: G449-L450, P451-N457, E463-R467	Chain A: S4
C_5	Chain A: S4-E37, E49-S61, V101-P106	/
C_6	Chain A: R47, G169-R174, S237-G241, V293-V295, D297-M308	Chain A: S2
C_7	Chain A: P48, K107-V113, M175-I196, R221-I223, A474-P482	/
C_8	Chain A: A201-S205	/
C_9	Chain A: V206-R220	/
C_{10}	Chain A: C231-S235	Chain A: S2
C_{11}	Chain A: L309-I319, V408-Y419, L494-T497	/
C_{12}	Chain A: A370-L373;<br>Chain B: V522	Chain A: S3
C_{13}	Chain A: S354-V356;	Chain A: S3
$C_{0'}$	Chain B: N66, Y70-R88, Y116-G118, S122-Y134, S145, E199, S200, L224, Q225-G227, L321-V323, N324-E327, F330, L420-A427, S428-W432, G437-L450, Y458-E462, R468, S490-F492, F502-H513, R515	Chain B: Ω-loop+S1+S2+S3+S4
$C_{1'}$	Chain B: I99-V101, P106-V113, W114, I115, A136-V144, W179-G198	Chain B: S1
$C_{2'}$	Chain B: F35-K51, T62-N65, C67-Q69, E89-C94, L95-N98, L146-V150, G151-Q178, M208-R220, S237-F284, S291-I296, D297-F301, S311, N313,	Chain B: Ω-loop+ S1+S2

	K315	
$C_{3'}$	Chain A: F523<br>Chain B: D285-F288, G328, S329, F331-V360, H362, A363, G367, L368, T372, Q374-W378, D381-V395, P433-M436, M520, W524	Chain B:S2+S3+ S4
$C_{4'}$	Chain A: W378-D381<br>Chain B: S228-C231, G396-V400, I401-H406, V408, K498-K501, Q514, L516-Q519	Chain A: S3<br>Chain B: S2+S3
$C_{5'}$	Chain B: S4-P34, K52-S61, P102-R105	/
$C_{6'}$	Chain B: S24, L135, P451-N457, E463-R467, I469-Y472	/
$C_{7'}$	Chain B: G119-Y121, R289, F290	Chain B: S1+S2
$C_{8'}$	Chain B: A201-G207	/
$C_{9'}$	Chain B: R221-I223, F314, K316-L320, F407, N409-Y419, W473-E489, P493-T497	/
$C_{10'}$	Chain B: P232-V236	Chain B: S2
$C_{11'}$	Chain B: P302-N310, G312	/
$C_{12'}$	Chain A: K530, N533<br>Chain B: P361, N364, L366, D369-V371, C521, F523, N525-P529, L531-A534	Chain B: S3

IV

#Community	Residues	Subdomains
C_0	Chain A: T62-N66, C67-M90, Y116, G118-V129, N131-L135, S145-Y148, E199, R243, K270-L282, F290-F292	Chain A: Ω-loop+S1+S2
C_1	Chain A: A136-V144	Chain A: S1
C_2	Chain A: G154-Q162, P232-R242, R244-L266, E268, K269	Chain A: S2
C_3	Chain A: G117, Y130, S200, Q225, S226, P283-R289, G322, V323, N324-M353, K357-A363, L368, V371, T372, Q374-D377, D380-V400, I401-H406, Y421-A427, S428-L450, P451-M470, S490, F502-Q514, L516-V522, N525<br>Chain B: L373	Chain A: S1+S2+S3+S4<br>Chain B: S3
C_5	Chain A: P106-I115, N183-G198, D217-R220, H471-T479	/
C_6	Chain A: D2-V8, V15-S28, W58-S61, P104, R105	/

**C_7**	Chain A: A29-K52, S91-C94, L95-S103, R149, V150, G151-F153, E163-A176, R267	ChainA: Ω-loop+ S1+ S2
**C_8**	Chain A: S354-V356	Chain A: S3
**C_9**	Chain A: L177, R216, R221-L224, V293-I296, D297-L321, F407-L420, L494-K501, R515	Chain A: S3
**C_{10}**	Chain A: A201-S215	/
**C_{11}**	Chain A: G227-C231	Chain A: S2
**C_{12}**	Chain A: N364, L366, G367, F523, W524, Q526-N533<br>Chain B: D369, A370	Chain A: S3<br>Chain B: S3
**C_{13}**	Chain A: N9-K14, P53-V57, Q178-D182	/
**C_{14}**	Chain A: G480-P493	/
**$C_{0'}$**	Chain B: T62-N66, C67-M90, L97, G117-V129, S145-Y148, E199, L224, R244, Q272-W279, P283, F284, F290-F292	Chain B: Ω-loop+ S1+ S2
**$C_{1'}$**	Chain B: A136-V144	Chain B: S1
**$C_{2'}$**	Chain B: P34-K51, S91-C94, L95, Y96, R149, V150, G151-L177, C231-R243, A245-P271, N280-L282, V293-I296, D297-E299	Chain B: Ω-loop+ S1+ S2
**$C_{3'}$**	Chain A: L373, W378, M379<br>Chain B: Y116, S200, A201, Q225-N230, D285-R289, L321-V323, N324-H362, N364, G367, L368, V371, T372, Q374-V400, I401-H406, L420-A427, S428-L450, P451, L452, E462-W473, K498-N525	Chain A: S3<br>Chain B: S2+S3+S4
**$C_{5'}$**	Chain B: P106-I115, Q178-G198	/
**$C_{6'}$**	Chain B: D2-S61, N98-R105, Y130-L135	Chain B: S1
**$C_{7'}$**	Chain B: G202-R220, G312-K315	/
**$C_{8'}$**	Chain B: R221-I223, K316-L320, V408-Y419, L494-T497	/
**$C_{9'}$**	Chain B: F300-S311, F407	/
**$C_{10'}$**	Chain B: V453-E461	/
**$C_{11'}$**	Chain B: A474-K478	/
**$C_{12'}$**	Chain A: D365, D369, A370, A534<br>Chain B: A363, D365, L366, Q526-A534	Chain A: S3<br>Chain B: S3
**$C_{13'}$**	Chain B: T479-P493	/
